# Development, Implementation, and Evaluation of a Personalized Machine Learning Algorithm for Clinical Decision Support: Case Study With Shingles Vaccination

**DOI:** 10.2196/16848

**Published:** 2020-04-29

**Authors:** Ji Chen, Sara Chokshi, Roshini Hegde, Javier Gonzalez, Eduardo Iturrate, Yin Aphinyanaphongs, Devin Mann

**Affiliations:** 1 Department of Population Health New York University School of Medicine New York, NY United States; 2 Medical Center Information Technology New York University Langone Health New York, NY United States; 3 Clinical Informatics New York University School of Medicine New York, NY United States

**Keywords:** clinical decision support, machine learning, alert fatigue, implementation science

## Abstract

**Background:**

Although clinical decision support (CDS) alerts are effective reminders of best practices, their effectiveness is blunted by clinicians who fail to respond to an overabundance of inappropriate alerts. An electronic health record (EHR)–integrated machine learning (ML) algorithm is a potentially powerful tool to increase the signal-to-noise ratio of CDS alerts and positively impact the clinician’s interaction with these alerts in general.

**Objective:**

This study aimed to describe the development and implementation of an ML-based signal-to-noise optimization system (SmartCDS) to increase the *signal* of alerts by decreasing the volume of low-value herpes zoster (shingles) vaccination alerts.

**Methods:**

We built and deployed SmartCDS, which builds personalized user activity profiles to suppress shingles vaccination alerts unlikely to yield a clinician’s interaction. We extracted all records of shingles alerts from January 2017 to March 2019 from our EHR system, including 327,737 encounters, 780 providers, and 144,438 patients.

**Results:**

During the 6 weeks of pilot deployment, the SmartCDS system suppressed an average of 43.67% (15,425/35,315) potential shingles alerts (appointments) and maintained stable counts of weekly shingles vaccination orders (326.3 with system active vs 331.3 in the control group; *P*=.38) and weekly user-alert interactions (1118.3 with system active vs 1166.3 in the control group; *P*=.20).

**Conclusions:**

All key statistics remained stable while the system was turned on. Although the results are promising, the characteristics of the system can be subject to future data shifts, which require automated logging and monitoring. We demonstrated that an automated, ML-based method and data architecture to suppress alerts are feasible without detriment to overall order rates. This work is the first alert suppression ML-based model deployed in practice and serves as foundational work in encounter-level customization of alert display to maximize effectiveness.

## Introduction

### Background and Significance

The potential effectiveness of clinical decision support (CDS) alerts as a scalable tool for promoting evidence-based care for vaccine administration has led to their frequent use by most health care systems seeking to maximize vaccination rates [1-4]. CDS alerts to prompt evidence-based practices have been extensively studied and shown to work best when delivered at an appropriate time and place in the clinical workflow, that is, when the clinician is prepared to receive the information [5-8]. Successful CDS alerts have led to a reduction in prescribing brand-name antibiotics [9], improved lipid management in renal transplant patients [10], improved compliance with guidelines for treating HIV [11-13], reduced ordering of tests when costs were displayed [14], and age-specific alerts that reduce inappropriate prescribing in the elderly [15-20].

Although CDS tools are effective reminders of best practices, their effectiveness is blunted by the context in which they are deployed; *alert fatigue* (clinician desensitization driven by overwhelming number and quality of safety alerts) [21] is the result of an ever-growing number of alerts in the electronic health record (EHR), leading to clinicians commonly ignoring or failing to respond appropriately to alerts. Alert fatigue resulting from an excess of poor-quality alerts (eg, alerts firing at inappropriate times or for inappropriate patients) contributes to clinicians’ perceptions that the bulk of alerts are likely clinically insignificant regardless of their clinical message. As a result, clinicians now override most medication alerts [22-24] and are becoming increasingly desensitized to alarms [25-27]. Although there is limited consensus on how to measure alert fatigue and its unintended consequences, data show that alert fatigue is significantly impacting the clinician experience and patient care [28-31]. At our large academic health system, the number of active interruptive alerts for providers grew from 13 in 2012 to 107 in 2018, an increase of more than 800%. In December 2018, our providers ordered the shingles vaccine in response to just 6.43% (2219/34,531) of the alerts, indicating that our clinicians view a majority of these alerts as inappropriate. Consequently, an improved EHR experience for clinicians has become an institutional priority for many health systems, including our own.

Individual-level factors, including clinicians’ bias toward ignoring alerts and poor signal detection resulting from the overwhelming number of alerts, and poor alert reliability add to the degraded effectiveness of CDS and user experience [32,33]. To optimize a CDS system means to optimize the *signal-to-noise* ratio of alerts by increasing the *signal*, decreasing the *noise* created by an abundance of inappropriate, poorly timed alerts or both. To this end, prior work in medication alerts and monitoring alarms have implemented advanced interventions that use rules to surface or suppress alerts, intending to improve CDS alert signal. A study using basic rules to deactivate irrelevant alerts and manually alter other alert frequencies based on severity decreased the override rates from 33.6 to 4.6 per 100 orders [34]. Similar severity ranking showed success in increasing alert acceptance rate by 50%, despite a 60% increase in alert events [35]. Other studies attempting to reduce noise, however, achieved limited or mixed results [36-45].

Research indicates that delivering alerts at the appropriate time and place in the clinical workflow is key to effective CDS [5-8]. Prior work to optimize CDS tools focuses on manual approaches [4,46]; these have proven to be time consuming, difficult to maintain, and static, limiting scalability. Optimization and incorporation of more sophisticated rules to surface or suppress alerts achieves limited reduction [36-41]. Machine learning (ML) is a powerful tool for identifying patterns in complex data by using past data to predict future performance. The use of ML in health care has proliferated over the past 10 years in a variety of use cases. ML applied to EHR data specifically shows signs of promise as a tool for improving safety and quality of care; its application to problems such as predicting readmission and sepsis shows the ability of ML ability to better target alerts to the appropriate user and use case [47-49]. An EHR-integrated ML algorithm is a potentially powerful tool to improve the quality of care by increasing the signal-to-noise ratio of alerts to positively impact clinicians’ interactions with these alerts. To date, the informatics literature lacks both prospective evaluation of signal-to-noise optimization interventions as well as detailed accounts of operational steps necessary to implement ML models in clinical care. Using the shingles vaccination alert as our initial use case, we leveraged historical EHR interaction data (clicks), patient and provider sociodemographic data to (1) build and train an ML model that can predict the likelihood of provider interaction with the shingles vaccination alert and (2) establish the data architecture necessary to deploy the model in a live environment.

### Objective

The objective of this case study was to describe the development, implementation, and prospective evaluation of a novel, ML-based, CDS signal-to-noise optimization (SmartCDS) system that suppresses low-value vaccination alerts applied to a shingles vaccination CDS alert.

## Methods

### Setting

This work was conducted within a large urban academic hospital system with approximately 1300 beds over several satellite locations. In the fiscal year 2016, 3584 doctors and 4899 nurses treated approximately 38,000 inpatient admissions, 5.8 million outpatient visits, and 150,000 emergency department visits.

### Data

**Figure 1:**
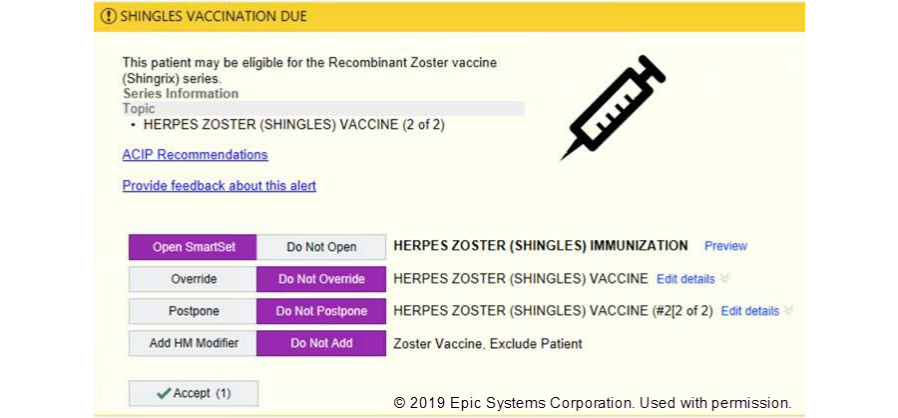
Screenshot of the shingles vaccination electronic health record alert.

This study uses all data from January 1, 2017, to March 11, 2019, to maintain consistency with the shingles alert content and its clinical setting. The dataset includes a total of 695,311 shingles alerts presented to 780 providers over 327,737 encounters, covering 144,438 unique patients. The overall alert interaction rate (any action toward acknowledging the shingles alert in an encounter) during this period was 16%, and the overall order rate of the shingles vaccine in response to the alert was 5%. The alert response options are illustrated in Figure 1—providers may choose from four different actions: *open SmartSet* to sign vaccine orders for targeted patients, health maintenance *override*, *postpone* or customize health maintenance modifier based on refusal, and deferral or other decisions made by patients; the alert appears on a side tab located at the right side of the EHR interface and may also be ignored or closed when no action is taken.

### Alert Suppression Model Construction

#### Feature Construction

A retrospective data query was performed to extract data related to the shingles vaccine alert. Key data elements to be extracted from the EHR were determined using a combination of descriptive analysis and clinical expertise. After data cleaning, historical changes were analyzed in alert usage to determine the optimal period from which data can be extracted for model training (two years of data from January 1, 2017, to December 31, 2018). Using data from our alert system, the average response rates for the alerts as well as the providers’ interaction history with the alerts were examined for the purpose of determining an appropriate protocol for assigning one unique provider to each alert encounter. Initial analyses demonstrated a large variation among clinicians with regard to the frequency of interaction with the alerts (0%-92%), prompting our team to construct variables for an individual clinician’s activity history, which was expanded to several short-term and long-term activity history variables capturing response rates, alert volume, and demographic variables for both clinicians and patients. The features that affect clinician’s response include (1) clinician-level demographics, clinical roles, and specialties; (2) response rate to previous shingles alert (both short-term and long-term); and (3) the number of recent encounters. The patient-level data included were patient demographics and history of targeted shingles alert responses and shingles vaccine orders by clinicians. In addition, a binary flag indicating walk-in visits and scheduled office visits was included as the architecture did not capture walk-in visits in our pilot implementation.

#### Machine Learning Model

**Figure 2:**
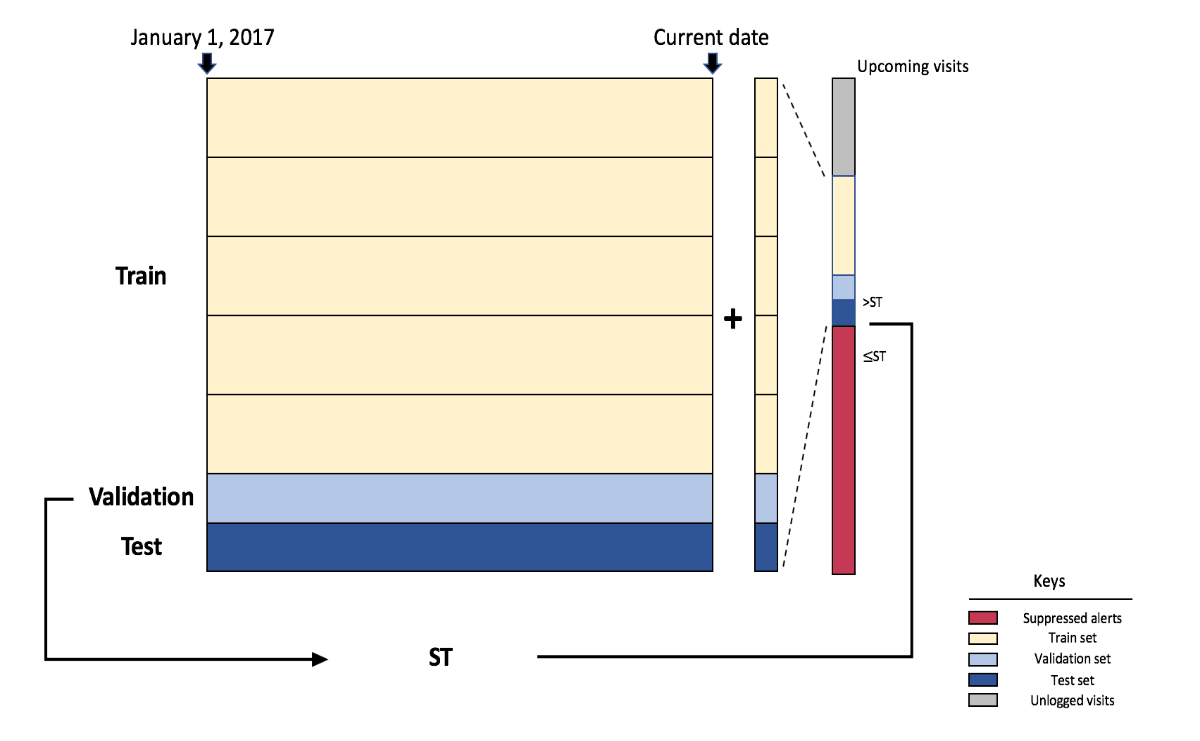
Experimental design. All data from January 1, 2017, to March 11, 2019, are used to train the model. Data are divided based on clinicians into 80%, 10%, 10% splits as train, validation, and test set, respectively. Each day, the data from the previous day are added to the dataset and the model is retrained and evaluated on the updated validation set to derive the 50% suppression score threshold. Predictions are made on upcoming visits (appointments) following the shingles best practice advisory (BPA) eligibility in the next day, and BPA instances are suppressed if the predicted score is lower than the threshold. Upcoming visits, which are logged into the shingles BPA log in the electronic health record system, are used for training in the future. In this design, the training window is always growing, with January 2017 as the start date. ST: score threshold.

The model was designed as a binary classification task. The target labels were built based on whether an alert instance was interacted with or whether a follow-up order for shingles vaccination was placed in each primary care visit. The data were split randomly based on individual clinicians into 80%, 10%, and 10% sets for model training, validation, and testing, respectively, as illustrated in Figure 2. XGBoost was employed as our ML algorithm, with learning rate=0.3, maximum tree depth=0.6, minimum child weight=1, no subsampling, negative log loss, and early stopping (with a maximum of 50 rounds). The validation set was used to monitor the model training through early stopping to derive the operational score threshold and evaluate the model performance; the test set was used to evaluate the effectiveness of the score threshold and the generalizability of the trained model retrospectively. To evaluate the performance of the model, we obtained a sample of nearly 65,000 primary care visits. We reported a highly effective model, adopting individual profiling of providers to reduce the number of clinically insignificant alerts, with average area under receiver operating characteristic of 0.919 and average area under precision-recall curve of 0.562 using 5-fold cross-validation. Our simulation found that of the 50.00% (6490/12,980) lowest ranked vaccination alerts, 99.77% (6475/6490) have been ignored by providers if not suppressed. Given that the corresponding estimated order reduction via nested cross-validation was deemed conservative at 1%, a 50% suppression threshold was selected in collaboration with clinical stakeholders [50]. As a result, the model that relies on personal history for features is updated daily to incorporate the latest data and update the 50% score threshold during prospective implementation. Upcoming appointments are used for ongoing training, making the training window ongoing. The patients’ appointments for initial primary care visits are excluded from suppression.

### Pilot Design and Evaluation

**Figure 3:**
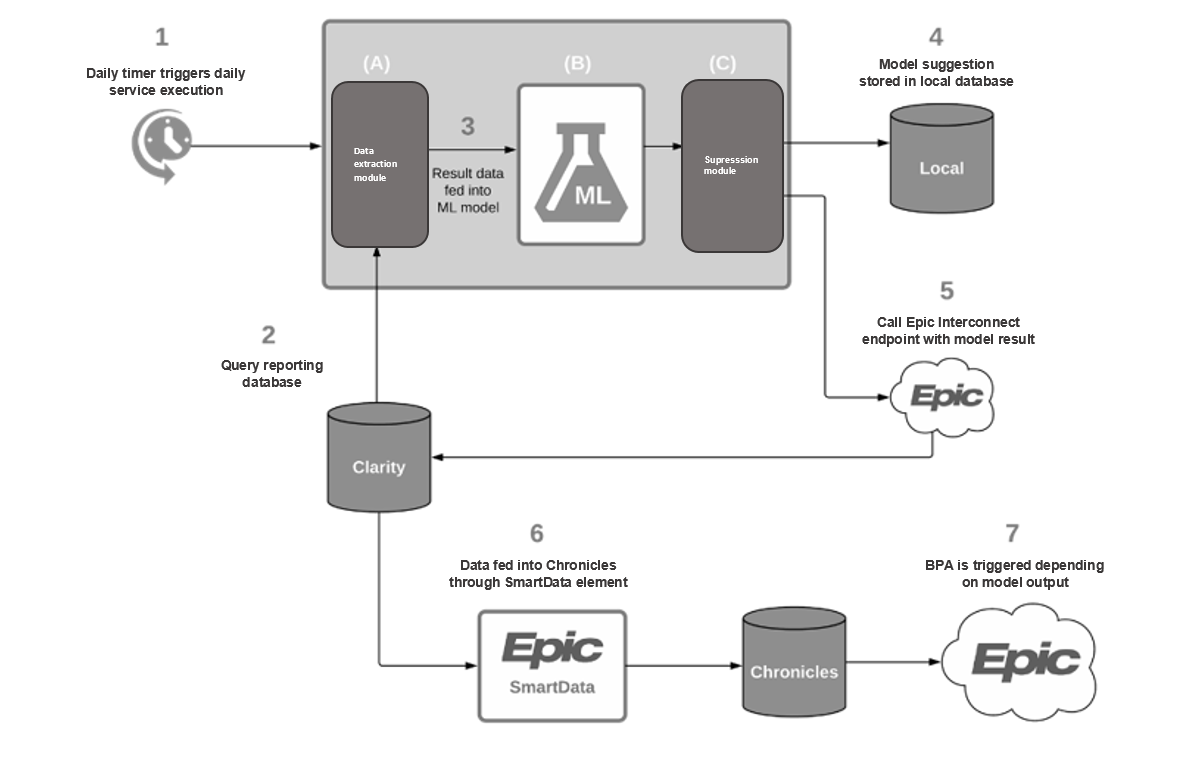
SmartCDS system architecture. This illustrates the SmartCDS machine learning (ML) implementation flow. (1) A timer (Cron job) is configured to run every day and invoke the SmartCDS service. (2) The data extraction module will query the reporting database (Epic Clarity) and feed (3) encounter, provider, user demographic, and best practice advisory data to the ML model. (4) The model output is then both stored in a local database for further analysis and pushed (5) to Epic through an Epic Interconnect Web Services endpoint. From here, information about what alert per encounter should be suppressed is written (6) into the Epic event database (Chronicles) through a SmartData element. An alert rule will inspect (7) these data to allow or suppress the alert being fired. BPA: best practice advisory; ML: machine learning.

A pilot study was designed over 6 weeks (Figure 3) in biweekly cycles (alternating turning the model on for one week and turning the model off for another week) to verify that the data distribution in the training/validation set was applicable to that in production. In the pilot, key statistical measures were examined with the model both turned on and turned off to compare prospective model performance with estimates generated in retrospective evaluation. The provider response and follow-up orders associated with suppressed shingles alerts cannot be measured; therefore, prospective model performance was evaluated using the percentage of daily suppressed alerts, daily alert response rate, weekly shingles vaccination order count, and alerts per order rate. Weekly aggregated measures were employed because of weekly patterns detected in the clinical setting (eg, Wednesdays and weekend days featured lower alert volume).

## Results

### Architecture for Signal-to-Noise Optimization System Deployment

After the construction of ML model for alert suppression (see Methods), we built a new data architecture to operationalize the model (Figure 3). The overall signal-to-noise optimization (SmartCDS) system was broken into three components: (1) the *data extraction module*, which identifies planned visits for the next day, with the intent to identify upcoming vaccine alerts to suppress, and queries the EHR to extract the variables required to run the ML module; (2) the suppression ML model itself (the *ML module* built as described above); and (3) the *suppression module*, which leverages a series of application programming interface calls to the EHR to communicate the alerts that should to be suppressed. The data extraction module queries the EHR and extracts features that should then be passed to the ML module.

The steps, related tasks, and timeline for the development and operationalization of the system are detailed in Table 1.

**Table 1:** Signal-to-noise optimization (SmartCDS) system development.

Step	Task	Timeline
Alert suppression model construction	Retrospective data query: Manually retrieved historical data (best practice advisory alert log, shingles vaccine order log, and patient/provider demographics) from our EHR^a^ databases Data cleaning and initial analysis: Aggregated data and conducted analyses to determine the average response rate for alerts Construction of predictive variables: Used long-term and short-term (1 month) personal interactive history of providers and clinician/patient demographics Model training and performance evaluation: Used constructed variables to build model and evaluated performance using a presplit (based on clinicians) training set (80%), validation set (10%), and test set (10%) from historical data Predictive variables and refinement of retrained models: Iterated predictive variable construction, model training, and performance evaluation on the validation set to determine the optimal predictive variables to train the model Optimization of model parameters: Optimized model performance by fine-tuning built-in model parameters	3 months
Aggregation of production data	Virtual table creation: Retrieved live data from our EHR databases (alert log, shingles vaccine order log, and patient/provider demographics) Storage of interim, preprocessed data on local database: Created repository to track results of each run (eg, errors)	2 weeks
Web service endpoint configuration	Web service isolation: Determined which Web service to call within our EHR’s interoperability Web Application Programing Interface Endpoint rule creation (part 1): Built rule that reacts to data sent to endpoint Suppression rule creation (part 2): Built rule that can determine whether to suppress the alert or not	3 weeks
Machine learning script optimization	Data query: Incorporated live data into model Feature engineering and storage to local database: Updated daily additive dataset as model is retrained with most recent log Model training: Trained model daily to incorporate live data Threshold setting: Used training and validation datasets to simulate the predicted relationship of alerts suppressed and orders missed Storage of model prediction results in local database: Recorded predictions, model scores, dates, and the corresponding score threshold for each upcoming vaccine alert	1 month
Docker image and container formation	Configuration of Web service setup: Installed required modules, packages, and drivers as well as tested Web service endpoint Cron job setup: Defined frequency and timing of specific system functions Logging: Monitored and recorded system function, including errors	1 week
Reporting and dashboard development	Production of relevant data elements and storage in local database: Daily report of summary statistics as tables and plots recorded Report delivery and cadence: Daily logging on status of the pipeline; email sent upon fatal errors	2 weeks

^a^EHR: electronic health record.

#### Aggregation of Production Data and Configuration of a Web Service Endpoint

With the alert suppression model built and data aggregated to support production, we worked with our institutional EHR team to create predefined and operationally approved queries to build easy-to-access views of our variables of interest in the EHR database. This enabled and automated the data extraction needed to operationalize the SmartCDS system. We then established a local database to serve as a repository for monitoring and tracking data runs, reports, and system errors per best practices. To complete our work on creating the technical capacity necessary to implement the SmartCDS system, we worked with the enterprise information technology team to determine the appropriate Web service to *call*; we then created the rules necessary to appropriately respond to the data sent to that endpoint and, if appropriate, suppress the target alert (in this case, the shingles vaccination alert).

#### Optimization of Machine Learning Script and Docker Image and Formation of Container

Once built, we validated the SmartCDS system with the shingles alert. Predictions are made on upcoming appointments by applying the model to our predefined views, modifying the ML script, and generating model predictions (suppress yes or no), which are saved in a local database and applied to suppress an alert with a predicted score less than the threshold (additional details in Methods). The system was designed to be modular and orthogonal with regard to call frequency, instrumentation, and configuration, allowing for easy adaptation to new environments. Under these parameters, we formed Docker containers (standard units of software that package code and all its dependencies, so each application runs quickly and reliably from one computing environment to another) to appropriately configure the Web service, define the frequency and timing of functionality, and log the system’s normal and error events.

#### Reporting Dashboard Development

**Figure 4:**
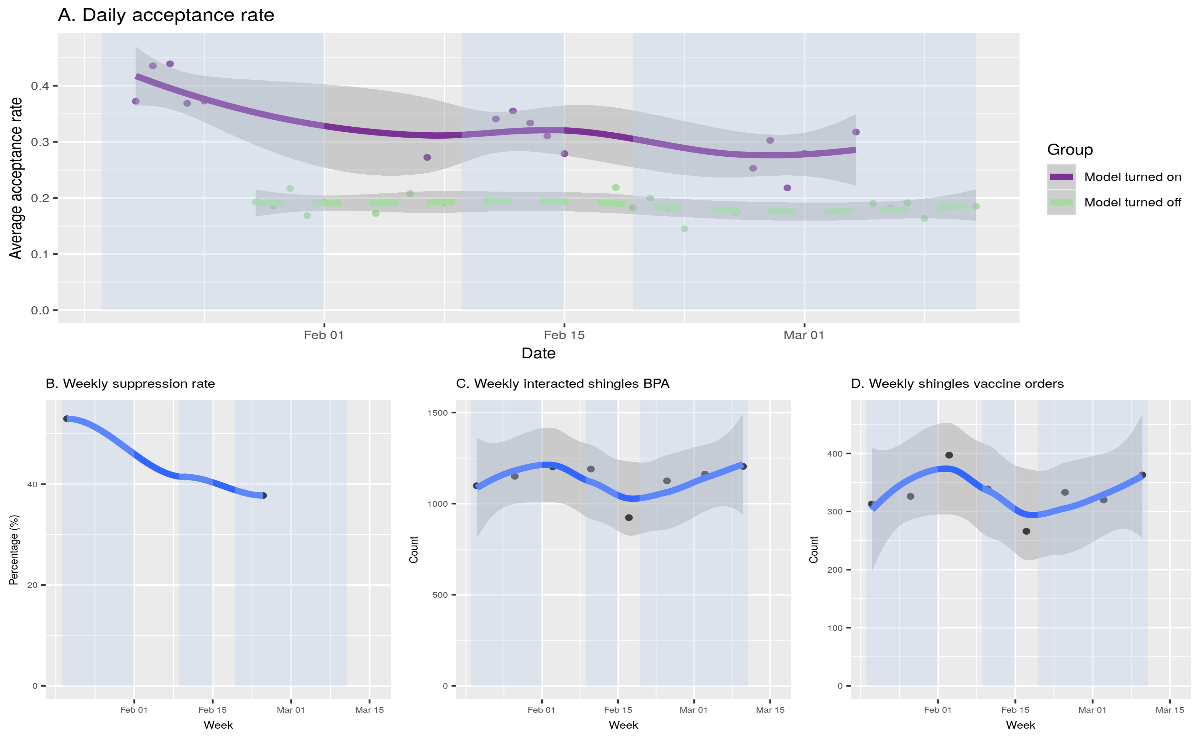
Summary reports of the shingles model from January 19, 2019, to March 11, 2019. (A) Smoothed curves of daily aggregated acceptance (response) rates to the shingles best practice advisory (BPA; alert), 95% CI shown in shaded areas, respectively (model turned on is shown in the purple solid curve and model turned off is shown in the light green dotted curve). Weekends are not included because of the large variation resulting from low BPA volumes on weekends. Vertical shaded areas annotate the 6-week trial time period. (B) Weekly averaged suppression percentage of the shingles alert. (C) Weekly count of interacted shingles BPA. (D) Smoothed curve of weekly shingles vaccine order counts.

A dashboard was developed to ensure that the system was running properly with a stable performance of the model from a safety and operational perspective and to monitor process outcomes of interest. The dashboard features process outcomes of interest (eg, suppression percentage, daily order counts, and daily alert volume) and factors in timing and frequency of report delivery based on feedback from clinical and operational stakeholders. Daily logging and weekly monitoring reports (Figure 4 and Multimedia Appendix 1) were constructed to enable detection of abnormal model behavior related to data shifts or model failures. If, on any date, the alert-related volume diverges from previous patterns, the alert log stream along with the related EHR data can be examined to locate the source of the anomaly.

### Pilot Results

#### Daily Alert–Related Volumes

We leveraged the 6 weeks of data (January 19-March 11, 2019) to compare the volume difference in shingles alert count, interacted alert count, and order count between weeks with the model turned on and turned off. We observed 42.2% (3541.0 with the model turned on vs 6123.7 with the model turned off) reduction in the alert count, no significant reduction in the interacted alert count (one-sided two sample *t* test; *P*=.20) or in the order count (one-sided two sample *t* test; *P*=.38) during the 6-week biweekly cycle with the model turned on and model turned off (Table 2).

**Table 2:** Shingles alert–related volume in 2019. Each statistic is the weekly average of the corresponding group during the 6-week biweekly cycle except that alerts per order rates were calculated as bulk averages within each 3-week group, respectively.

Group	Time range (weeks)	Alert count, mean (SD)	Interacted alert count^a^, mean (SD)	Order count^b^, mean (SD)	Accumulated alerts per order rate	Accumulated interacted alerts per order rate
Model turned off	3	6123.7 (232.9)	1162.3 (22.8)	331.3 (5.1)	18.5	3.4
Model turned on	3	3541.0 (669.4)	1118.3 (71.6)	326.3 (23.6)	10.9	3.5

^a^*P=*.20.

^b^*P=*.38.

#### Alerts per Order Rate and Signal-to-Noise Ratio

Our 6-week pilot deployment of the system in the live environment indicates an alert suppression rate of 43.7% out of 35,315 appointments (Figure 4), with stable shingles vaccine order volume (no statistically significant difference between active and inactive suppression) slightly lower than the predefined 50% threshold. Initial inspection showed that, on an average, walk-in visits had a higher alert ignored rate (91%) compared with scheduled office visits (87%) in 2017 and 2018. As the model only operated on scheduled appointments in this study and the activity history has the highest weight toward a suppression decision, a slightly lower suppression rate than 50% was expected.

The ratio of alerts fired to orders placed with the model turned on was almost half of that of the ratio with the model turned off, whereas the ratio of the interacted alerts per order placed remained the same. By mapping the average orders placed as the average power of *signal* and the average count of ignored alert with no follow-up orders as the average power of *noise*, the signal-to-noise ratio changed from 5.7% to 10.1%, a 78% increase. Furthermore, by mapping the interacted alerts (including follow-up orders) as *signal* and the ignored alerts with no follow-up actions as *noise*, the signal-to-noise ratio changed from 23.4% to 46.1%, a 97% increase.

## Discussion

### Principal Findings

This paper describes the steps and considerations involved in the development and implementation of an ML model for suppressing low-value alerts in the EHR for the shingles vaccination. As predicted in our simulation, validation of this signal-to-noise optimization (SmartCDS) system demonstrated substantial reduction in the shingles vaccine alerts at a limited vaccine ordering expense. The rate of daily alert interaction among individual clinicians during the 6-week pilot was higher with the model turned on vs the model turned off. This result was expected because of the 42.2% lower volume of shingles alerts observed with stable daily alert interactions. Interestingly, the overall interaction rate gradually decreased over the 6-week cycle (Figure 4). This finding is consistent with the findings that responsiveness to alerts tends to decrease over time [29]. During the 6-week pilot, the profile of the providers who accepted the alerts did not change, indicating that the profile of patients who are offered the vaccination did not change either. This will be confirmed in our follow-up studies. To date, our literature review indicates that our SmartCDS system is the first to develop an ML-based system to suppress clinically insignificant alerts or alerts unlikely to be accepted and to prospectively evaluate the system in a large-scale health care system. Relevant literature to date has been limited to retrospective studies focused on identifying false-positive or clinically insignificant physiologic monitor alarms (false alarms). In 2015, Physionet opened a challenge to reduce false arrhythmia alarms using a subset of the Medical Information Mart for Intensive Care II waveform database [51]. The best models showed that by allowing 30 seconds of delay, false alarms can be better distinguished from true alarms; the best models were able to achieve 80% reduction in false alarms, missing 1% of true alarms. Studies focusing on pulse oximetry to reduce peripheral capillary oxygen saturation (SpO_2_) false alarms, intracranial pressure alarms, and general vital sign monitoring alarms found mixed results ranging from 25% to 47% in alarm reduction, with 0% to 5% false-negative rates [42-45]. A more recent study showed that, by increasing delayed time within 3 min for alarms with physiologic monitoring waveforms, as well as including electrocardiography, SpO_2_, and arterial blood pressure, an ML model can achieve slightly better performance but fails to stably generalize to unseen data [52].

The development of a robust reporting structure allows for the logging and monitoring of the system and its impact on clinical outcomes, which are necessary to ensure the stability and safety of the system. Future work will involve gathering feedback from front-line stakeholders to support the adaptation of the signal-to-noise optimization system to other alerts, enabling the system to ingest real-time data as well as further development of a reporting dashboard with effective, user-centered data displays, and a systematic process for establishing organizationally acceptable thresholds for alert suppression.

### Limitations

During the pilot implementation and evaluation, the model only operated on scheduled office visits because of infrastructure gaps restricting the ability to incorporate walk-in visits. We are working to address this gap to be able to assess the effectiveness and impact of this model on a global level. On the other hand, it is possible that clinicians will start to adjust to the volume change in the shingles alert delivery, leading to less responsiveness and less ordering. As potential external or systematic biases, such as seasonal effects, could lead to inaccurate observations and conclusions, we will implement a more comprehensive statistical evaluation after updating the infrastructure to systematically address these potential biases.

### Conclusions

Our model presented high discriminatory power in the initial prospective evaluation of shingles alert interactions. Our approach was effective in suppressing unnecessary alerts, with limited reduction in overall order volume. This work also provides potential evidence of increase in interactions and orders (eg, an increase in signal-to-noise ratio) by decreasing noise (eg, suppression). In addition, the process built to operationalize this new ML tool may prove to be a useful model for enabling the deployment of this type of tool across many use cases. Future efforts include applying this approach globally to other EHR alerts and comprehensive randomized controlled trials.

## Appendix

Multimedia Appendix 1 Daily count of the shingles best practice advisories (BPAs; alerts), interacted shingles BPAs, and shingles BPA follow-up vaccine orders from January 19, 2019, to March 11, 2019. Each bar in all three plots represents one day - purple (dark) if the model was turned on and light green with a black border if the model was turned off, the shaded areas annotate excluded dates from downstream statistical analysis. BPAL best practice advisory.

## Abbreviations

CDS: clinical decision support
EHR: electronic health record
ML: machine learning
SpO_2_: peripheral capillary oxygen saturation

## References

[1] McDonald CJ, Hui SL, Tierney WM (1992). Effects of computer reminders for influenza vaccination on morbidity during influenza epidemics. MD Comput.

[2] Dexter PR, Perkins S, Overhage JM, Maharry K, Kohler RB, McDonald CJ (2001). A computerized reminder system to increase the use of preventive care for hospitalized patients. N Engl J Med.

[3] Kim DK, Riley LE, Hunter P, Advisory Committee on Immunization Practices (2018). Recommended immunization schedule for adults aged 19 years or older, United States, 2018. Ann Intern Med.

[4] Osheroff JA, Teich JM, Middleton B, Steen EB, Wright A, Detmer DE (2007). A roadmap for national action on clinical decision support. J Am Med Inform Assoc.

[5] Hunt DL, Haynes RB, Hanna SE, Smith K (1998). Effects of computer-based clinical decision support systems on physician performance and patient outcomes: a systematic review. J Am Med Assoc.

[6] Overhage JM, Tierney WM, Zhou X, McDonald CJ (1997). A randomized trial of 'corollary orders' to prevent errors of omission. J Am Med Inform Assoc.

[7] Dexheimer JW, Talbot TR, Sanders DL, Rosenbloom ST, Aronsky D (2008). Prompting clinicians about preventive care measures: a systematic review of randomized controlled trials. J Am Med Inform Assoc.

[8] Sittig DF, Wright A, Osheroff JA, Middleton B, Teich JM, Ash JS, Campbell E, Bates DW (2008). Grand challenges in clinical decision support. J Biomed Inform.

[9] Bernstein SL, Whitaker D, Winograd J, Brennan JA (2005). An electronic chart prompt to decrease proprietary antibiotic prescription to self-pay patients. Acad Emerg Med.

[10] Garthwaite EA, Will EJ, Bartlett C, Richardson D, Newstead CG (2004). Patient-specific prompts in the cholesterol management of renal transplant outpatients: results and analysis of underperformance. Transplantation.

[11] Safran C, Rind DM, Davis RM, Currier J, Ives D, Sands DZ, Slack WV, Makadon H, Cotton D (1993). An electronic medical record that helps care for patients with HIV infection. Proc Annu Symp Comput Appl Med Care.

[12] Safran C, Rind D, Davis R, Ives D, Sands D, Currier J, Slack W, Makadon H, Cotton D (1995). Guidelines for management of HIV infection with computer-based patient's record. Lancet.

[13] Safran C, Rind D, Sands D, Davis R, Wald J, Slack W (1996). Development of a knowledge-based electronic patient record. MD Comput.

[14] Tierney WM, Miller ME, McDonald CJ (1990). The effect on test ordering of informing physicians of the charges for outpatient diagnostic tests. N Engl J Med.

[15] Simon S, Smith D, Feldstein A, Perrin N, Yang X, Zhou Y, Platt R, Soumerai SB (2006). Computerized prescribing alerts and group academic detailing to reduce the use of potentially inappropriate medications in older people. J Am Geriatr Soc.

[16] Shah N, Seger A, Seger D, Fiskio JM, Kuperman GJ, Blumenfeld B, Recklet EG, Bates DW, Gandhi TK (2006). Improving acceptance of computerized prescribing alerts in ambulatory care. J Am Med Inform Assoc.

[17] Tamblyn R, Huang A, Perreault R, Jacques A, Roy D, Hanley J, McLeod P, Laprise R (2003). The medical office of the 21st century (MOXXI): effectiveness of computerized decision-making support in reducing inappropriate prescribing in primary care. Can Med Assoc J.

[18] Gaikwad R, Sketris I, Shepherd M, Duffy J (2007). Evaluation of accuracy of drug interaction alerts triggered by two electronic medical record systems in primary healthcare. Health Informatics J.

[19] Smith DH, Perrin N, Feldstein A, Yang X, Kuang D, Simon SR, Sittig DF, Platt R, Soumerai SB (2006). The impact of prescribing safety alerts for elderly persons in an electronic medical record: an interrupted time series evaluation. Arch Intern Med.

[20] Seidling HM, Schmitt SP, Bruckner T, Kaltschmidt J, Pruszydlo MG, Senger C, Bertsche T, Walter-Sack I, Haefeli WE (2010). Patient-specific electronic decision support reduces prescription of excessive doses. Qual Saf Health Care.

[21] Hussain MI, Reynolds TL, Zheng K (2019). Medication safety alert fatigue may be reduced via interaction design and clinical role tailoring: a systematic review. J Am Med Inform Assoc.

[22] Isaac T, Weissman JS, Davis RB, Massagli M, Cyrulik A, Sands DZ, Weingart SN (2009). Overrides of medication alerts in ambulatory care. Arch Intern Med.

[23] van der Sijs H, Aarts J, Vulto A, Berg M (2006). Overriding of drug safety alerts in computerized physician order entry. J Am Med Inform Assoc.

[24] Carspecken CW, Sharek PJ, Longhurst C, Pageler NM (2013). A clinical case of electronic health record drug alert fatigue: consequences for patient outcome. Pediatrics.

[25] Hu X, Sapo M, Nenov V, Barry T, Kim S, Do DH, Boyle N, Martin N (2012). Predictive combinations of monitor alarms preceding in-hospital code blue events. J Biomed Inform.

[26] Voepel-Lewis T, Parker ML, Burke CN, Hemberg J, Perlin L, Kai S, Ramachandran SK (2013). Pulse oximetry desaturation alarms on a general postoperative adult unit: a prospective observational study of nurse response time. Int J Nurs Stud.

[27] Bonafide CP, Lin R, Zander M, Graham CS, Paine CW, Rock W, Rich A, Roberts KE, Fortino M, Nadkarni VM, Localio AR, Keren R (2015). Association between exposure to nonactionable physiologic monitor alarms and response time in a children's hospital. J Hosp Med.

[28] Korniewicz DM, Clark T, David Y (2008). A national online survey on the effectiveness of clinical alarms. Am J Crit Care.

[29] Embi PJ, Leonard AC (2012). Evaluating alert fatigue over time to EHR-based clinical trial alerts: findings from a randomized controlled study. J Am Med Inform Assoc.

[30] Genco EK, Forster JE, Flaten H, Goss F, Heard KJ, Hoppe J, Monte AA (2016). Clinically inconsequential alerts: the characteristics of opioid drug alerts and their utility in preventing adverse drug events in the emergency department. Ann Emerg Med.

[31] Joint Commission (2013). Medical device alarm safety in hospitals. Sentinel Event Alert.

[32] McCoy A, Thomas E, Krousel-Wood M, Sittig D (2014). Clinical decision support alert appropriateness: a review and proposal for improvement. Ochsner J.

[33] Ong M, Coiera E (2011). Evaluating the effectiveness of clinical alerts: a signal detection approach. AMIA Annu Symp Proc.

[34] Simpao AF, Ahumada LM, Desai BR, Bonafide CP, Gálvez JA, Rehman MA, Jawad AF, Palma KL, Shelov ED (2015). Optimization of drug-drug interaction alert rules in a pediatric hospital's electronic health record system using a visual analytics dashboard. J Am Med Inform Assoc.

[35] Cornu P, Steurbaut S, Gentens K, van de Velde R, Dupont AG (2015). Pilot evaluation of an optimized context-specific drug-drug interaction alerting system: A controlled pre-post study. Int J Med Inform.

[36] Paterno MD, Maviglia SM, Gorman PN, Seger DL, Yoshida E, Seger AC, Bates DW, Gandhi TK (2009). Tiering drug-drug interaction alerts by severity increases compliance rates. J Am Med Inform Assoc.

[37] Beccaro MA, Villanueva R, Knudson KM, Harvey EM, Langle JM, Paul W (2010). Decision support alerts for medication ordering in a computerized provider order entry (CPOE) system: a systematic approach to decrease alerts. Appl Clin Inform.

[38] Guzek M, Zorina OI, Semmler A, Gonzenbach RR, Huber M, Kullak-Ublick GA, Weller M, Russmann S (2011). Evaluation of drug interactions and dosing in 484 neurological inpatients using clinical decision support software and an extended operational interaction classification system (Zurich Interaction System). Pharmacoepidemiol Drug Saf.

[39] Czock D, Konias M, Seidling HM, Kaltschmidt J, Schwenger V, Zeier M, Haefeli WE (2015). Tailoring of alerts substantially reduces the alert burden in computerized clinical decision support for drugs that should be avoided in patients with renal disease. J Am Med Inform Assoc.

[40] Eppenga WL, Derijks HJ, Conemans JM, Hermens WA, Wensing M, de Smet PA (2012). Comparison of a basic and an advanced pharmacotherapy-related clinical decision support system in a hospital care setting in the Netherlands. J Am Med Inform Assoc.

[41] Harinstein LM, Kane-Gill SL, Smithburger PL, Culley CM, Reddy VK, Seybert AL (2012). Use of an abnormal laboratory value-drug combination alert to detect drug-induced thrombocytopenia in critically Ill patients. J Crit Care.

[42] Connor C, Gohil B, Harrison M (2009). Triggering of systolic arterial pressure alarms using statistics-based versus threshold alarms. Anaesthesia.

[43] Borowski M, Siebig S, Wrede C, Imhoff M (2011). Reducing false alarms of intensive care online-monitoring systems: an evaluation of two signal extraction algorithms. Comput Math Methods Med.

[44] Scalzo F, Hu X (2013). Semi-supervised detection of intracranial pressure alarms using waveform dynamics. Physiol Meas.

[45] Schmid F, Goepfert MS, Franz F, Laule D, Reiter B, Goetz AE, Reuter DA (2017). Reduction of clinically irrelevant alarms in patient monitoring by adaptive time delays. J Clin Monit Comput.

[46] van de Velde S, Heselmans A, Delvaux N, Brandt L, Marco-Ruiz L, Spitaels D, Cloetens H, Kortteisto T, Roshanov P, Kunnamo I, Aertgeerts B, Vandvik PO, Flottorp S (2018). A systematic review of trials evaluating success factors of interventions with computerised clinical decision support. Implement Sci.

[47] Futoma J, Morris J, Lucas J (2015). A comparison of models for predicting early hospital readmissions. J Biomed Inform.

[48] Mao Q, Jay M, Hoffman JL, Calvert J, Barton C, Shimabukuro D, Shieh L, Chettipally U, Fletcher G, Kerem Y, Zhou Y, Das R (2018). Multicentre validation of a sepsis prediction algorithm using only vital sign data in the emergency department, general ward and ICU. BMJ Open.

[49] Osheroff JA (2009). Improving Medication Use and Outcomes with Clinical Decision Support: A Step by Step Guide.

[50] Chen J, Aphinyanaphongs Y, Mann D, Iturrate E, Chokshi S, Hegde R (2019). Personalized Clinical Decision Support (CDS) using Machine Learning: A Case Study with Shingles. Proceedings of the AMIA 2019 Clinical Informatics Conference.

[51] Clifford G, Silva I, Moody B, Li Q, Kella D, Shahin A, Kooistra T, Perry D, Mark RG (2015). The PhysioNet/Computing in Cardiology Challenge 2015: Reducing False Arrhythmia Alarms in the ICU. Comput Cardiol (2010).

[52] Chen L, Dubrawski A, Wang D, Fiterau M, Guillame-Bert M, Bose E, Kaynar AM, Wallace DJ, Guttendorf J, Clermont G, Pinsky MR, Hravnak M (2016). Using supervised machine learning to classify real alerts and artifact in online multisignal vital sign monitoring data. Crit Care Med.

