# Supplementary figures and images for: Development, Implementation, and Evaluation of a Personalized Machine Learning Algorithm for Clinical Decision Support: Case Study With Shingles Vaccination
Source: J Med Internet Res. 2020 Apr 29;22(4):e16848. doi: 10.2196/16848 (PMC7221637; doi:10.2196/16848)

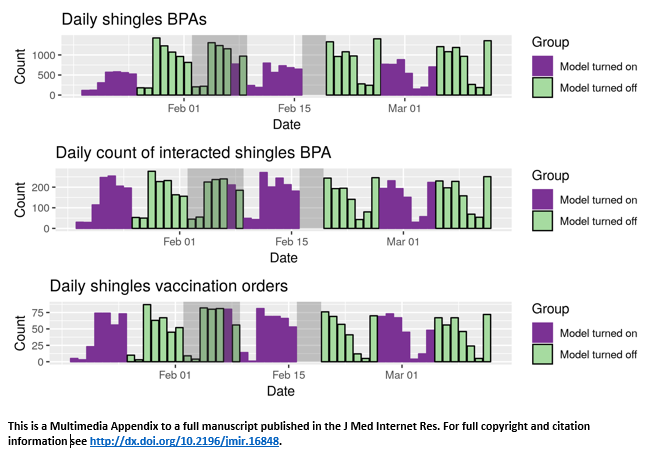

Supplement: Multimedia Appendix 1 [file jmir_v22i4e16848_app1.PNG]
